# *Adonis fucensis* (*A.* sect. *Adonanthe*, Ranunculaceae), a New Species from the Central Apennines (Italy)

**DOI:** 10.3390/biology12010118

**Published:** 2023-01-11

**Authors:** Fabio Conti, Christoph Oberprieler, Marco Dorfner, Erik Schabel, Roxana Nicoară, Fabrizio Bartolucci

**Affiliations:** 1 Floristic Research Center of the Apennine, University of Camerino—Gran Sasso Laga National Park, San Colombo, 67021 Barisciano, Italy; 2 Evolutionary and Systematic Botany Group, Institute of Plant Biology, University of Regensburg, Universitätsstr. 31, D-93053 Regensburg, Germany; 3 Department of Taxonomy, Ecology and Nature Conservation, Institute of Biology Bucharest—Romanian Academy, Splaiul Independenței 296, District 6, 060031 Bucharest, Romania

**Keywords:** *Adonanthe*, endemism, molecular phylogeny, nomenclature, taxonomy, steppic plant

## Abstract

**Simple Summary:**

*Adonis* sect. *Adonanthe* is characterized by species with strongly gibbous abaxial side of achenes, reticulate-venation on its surface, with short and recurved style and includes four series: ser. *Amurenses*, ser. *Coeruleae*, ser. *Apenninae*, ser. *Vernales*. In the Euro-Mediterranean area three species belonging to *A.* sect. *Adonanthe* are currently recognized: *A. apennina* (ser. *Apenninae*), *A. volgensis* (incl. *A. transsilvanica*; ser. *Vernales*), *A. vernalis* (ser. *Vernales*). In 2021 was discovered in the Central Apennines (Italy) a yellow-flowered *Adonis* population belonging to sect. *Adonanthe* similar to *A. volgensis*. Following an integrated taxonomic approach, we have shown that the newly discovered population should be regarded as a new species, named *A. fucensis*, endemic to Abruzzo (Central Apennines, Italy).

**Abstract:**

*Adonis fucensis* is herein described as a new species based on morphological and molecular analyses. It is endemic to one locality of the Central Apennines between Amplero and Fucino plains within the NATURA 2000 network in the SAC IT7110205 (Central Italy). The only discovered population is composed of 65 individuals and is at risk of extinction. The conservation status assessment according to IUCN categories and criteria is proposed and discussed. The new species belongs to *A.* sect. *Adonanthe* and is morphologically similar to *A. volgensis* (incl. *A. transsilvanica*), a species distributed in Hungary, Romania, Bulgaria, and Turkey as well as eastward to SW Siberia and Central Asia. *Adonis fucensis* can be distinguished from *A. volgensis* by larger cauline leaves, pentagonal with lobes lanceolate, larger stipules with more lobes and teeth, and larger flowers. Finally, an analytical key to *Adonis* species belonging to sect. *Adonanthe* distributed in Europe is presented.

## 1. Introduction

The genus *Adonis* L. (Ranunculaceae) comprises 38 accepted, annual and perennial, species and subspecies, distributed in the northern hemisphere and native to Asia, Europe, northern Africa, and Mediterranean region [1]. According to Wang [2,3], based on a morphological study, the genus *Adonis* should be divided into two subgenera, six sections, and six series: subg. *Adonis* (divided into three sections and two series) and subg. *Adonanthe* (Spach) W.T.Wang (divided into three sections and four series). Recent molecular studies [4,5] do not fully support the taxonomic treatment based on morphological features proposed by Wang [2,3], whereas a phylogenetic classification has not yet been established. In Italy, the genus *Adonis* is represented by 10 taxa (species and subspecies): the annual and red-flowered *A. annua* L., *A. flammea* Jacq. (with two subspecies), *A. aestivalis* L. (with two subspecies), *A. microcarpa* DC., and the perennial and yellow-flowered *A. distorta* Ten. and *A. vernalis* L. *Adonis distorta* is the only species of the genus endemic to Italy [6,7], growing in the alpine belt on limestone screes and less frequently on more stabilized rocky slopes between 1845 and 2675 m a.s.l. of the Central Apennines [8]. The Abruzzo administrative region, in Central Italy, hosts seven *Adonis* taxa, the highest number among the Italian administrative regions, including the rare endemic *A. distorta*, and the only Italian populations of the steppe species *A. vernalis* [9]. In recent years extensive field surveys have been carried out for floristic and vegetation research in the National Park of Abruzzo, Lazio, and Molise [10,11,12,13,14], thanks to which some plants typical of the Alpine continental valleys or even of the E-European steppes have been discovered (i.e., [15,16]), confirming that the inner basins of the Central Apennine mountains have a pronounced steppic character. In March 2021 a group of hikers, discovered within the buffer area of the National Park, a yellow-flowered *Adonis* population. After analyzing the photographic material sent to us by Marina Buschi, who discovered the plant, we immediately realized that we were dealing with a very interesting population of *Adonis* morphologically very different from the two yellow-flowered species that already occurred in Italy, which are *A. vernalis* and *A. distorta*. From March to June 2021, we performed field surveys in the discovery locality, close to Amplero in Collelongo municipality (L’Aquila, Abruzzo, Central Italy), to evaluate the numerical consistency of the population and look for new stations. According to the classification proposed by Wang [2,3], the new discovered *Adonis* population belongs to *A.* sect. *Adonanthe* W.T.Wang ser. *Vernales* Bobr. ex Poschk. The section is characterized by species with strongly gibbous abaxial side of achenes, reticulate-venation on its surface, with short and recurved style and includes four series [2,3]: ser. *Amurenses* Poschk. with petiolate lower cauline leaves, ovoid, triangular or elliptic, and yellow or white petals; ser. *Coeruleae* Poschk. with petiolate lower cauline leaves, oblong or ovoid-oblong, 3–4 pinnatisect, and white or purple petals; ser. *Apenninae* Bobr. ex Poschk. with sessile or sub-sessile cauline leaves, pinnately compound, segments 2–3 pinnatisect, and yellow petals; ser. *Vernales* with sessile cauline leaves, palmately compound, segments 3 pinnatisect, and yellow petals. In the Euro-Mediterranean area three species belonging to *A.* sect. *Adonanthe* are currently recognized [1,17]: *A. apennina* L. (ser. *Apenninae*), *A. volgensis* DC. (incl. *A. transsilvanica* Simonov.; ser. *Vernales*), and *A. vernalis* L. (ser. *Vernales*). All other currently accepted species [1] within *A.* sect. *Adonanthe* are distributed exclusively in Asia: *A. amurensis* Regel & Radde (ser. *Amurenses*), *A. davidi* Franch. (ser. *Amurenses*), *A. multiflora* Nishikawa & Koji Ito (ser. *Amurenses*), *A. pseudoamurensis* W.T.Wang (ser. *Amurenses*), *A. ramosa* Franch. (ser. *Amurenses*), *A. shikokuensis* Nishikawa & Koji Ito (ser. *Amurenses*), *A. sutchuenensis* Franch. (ser. *Amurenses*), *A. coerulea* Maxim. (ser. *Coeruleae*), *A. bobroviana* Simonov. (ser. *Apenninae*), *A. mongolica* Simonov. (ser. *Apenninae*), *A. tianschanica* (Adolf) Lipsch. (ser. *Apenninae*), *A. turkestanica* (Korsh.) Adolf (ser. *Apenninae*), and *A. villosa* Ledeb. (ser. *Apenninae*).

The closest species within *A.* sect. *Adonanthe* ser. *Vernales* based on morphology is *A. volgensis*, a typical plant of the E-European and Asiatic steppes, distributed in Hungary, Romania, Bulgaria, and Turkey, as well as eastward to SW Siberia and Central Asia [1,17,18]. The populations from Romania and Hungary were regarded by Wang [3] as a different species with the name *A. transsilvanica* Simonov. Instead, others authors have considered *A. transsilvanica* as synonym of *A. volgensis* (i.e., [1]), as a name ambiguous [19], or have not listed it at all [17,18].

An extensive morphological and molecular investigation has been carried out providing evidence about the differentiation between *A. volgensis* and the new discovered Apennines’ population. Our results, and the disjunct and isolated geographical distribution of the population occurring in the Central Apennines, allowed us to describe it as a species new to science, named *A. fucensis*.

## 2. Materials and Methods

### 2.1. Plant Material

This study is based on field surveys, an extensive analysis of the relevant literature, and examination of herbarium specimens (including the original material of *A. volgensis* and *A. transsilvanica*; Supplementary File S1) kept in APP, B, BP, BRNU, CL, G, K, LD, LE, MW, S, UPS, and US (acronyms follow [20]).

Morphological characters, recognized as taxonomically discriminant in *Adonis* [2,3,18,21] were scored in the herbarium specimens kept in APP, BP, CL, K, MW, UPS, and US. All morphological characters were observed and measured under a Leica MZ16 stereoscopic microscope, using a digital caliper with 0.1 mm precision. Digital images of herbarium specimens from online databases were measured with IC Measure version 2.0.0.245.

Regarding the new species, having found only one small population, we have not collected whole individuals, but only some parts, such as petals, sepals, leaves, etc., and then dried them. Only two individuals, without rhizomes, probably displaced by wild fauna, were collected. For the description of the new species some characters, i.e., length of the sepals and petals, were also scored in the field on fresh material.

### 2.2. Morphometric Analyses

A total of 18 morphological characters were selected and scored in 87 dried individuals belonging to *A. volgensis* (63) from Romania, Moldavia, Russia and to the new population from the Central Apennines (Italy), named *A. fucensis* (24). Two characters, i.e., height (H) and number of petals (NP), were scored for *A. fucensis* on the field. Among the morphological characters studied, 14 are quantitative, 1 is calculated ratio, and 3 are qualitative (Table 1). Samples with missing data were not included in the multivariate analysis (resulting dataset of 50 individuals × 18 variables). For each quantitative character an independent sample *t*-test was carried out with SPSS v25 software (IBM Corp., Armonk, NY, USA) [22]. A non-metric multidimensional scaling (NMDS) and Cluster Analysis (CA) using the average linkage method (UPGMA), were performed with PAST package v4.11 software (Natural History Museum, Oslo, Norway) [23]. The similarity matrix was calculated using the Gower coefficient, suitable for mixed data [24]. Furthermore, the variability of the analyzed morphological characters was described by standard statistical parameters (mean, standard deviation, minimum, maximum, and 25th and 75th percentiles). Boxplots were built through SPSS v25.

### 2.3. DNA Sequencing and AFLPseq Fingerprinting

Genomic DNA for both sequencing and genetic fingerprinting was extracted according to the CTAB DNA extraction protocol of Doyle and Dickson [25] and Doyle and Doyle [26]. Amplification of the two internal transcribed spacer regions (ITS1, ITS2) of the nuclear ribosomal repeat (nrDNA) was carried out with primers ITS-18SF [27] and ITS2 [28] for ITS1 and ITS-D [29] and ITS-SR [30] for ITS2, respectively. After purification of PCR amplicons with AmpliClean (Nimagen, Nijmegen, The Netherlands) magnetic beads, Sanger sequencing was carried out by a contract sequencing company (Macrogen Europe, Amsterdam, The Netherlands). Electropherograms were manually edited with CHROMAS v2.6.6 [31]; polymorphisms observed in accession A1251 were resolved manually and the two resulting sequences were independently included in the alignment together with sequences of other species of *Adonis* sect. *Adonanthe* and an outgroup sequence (from *Trollius ranunculoides* Hemsl.). We used PAUP* v4.0a169 [32] to calculate distances among the aligned sequences based on the Kimura-2-Parameter model and constructed a Neighbor-joining tree. A bootstrap analysis was performed with 1000 replicates.

The AFLPseq fingerprinting method has been proposed by [33] and combines the genome-complexity reducing AFLP approach [34] with the next-generation sequencing (NGS) of resulting AFLP bands using the Nanopore sequencer MinION from Oxford Nanopore Technologies (Oxford, UK). It provides sequence and single-nucleotide polymorphism (SNP) information for hundreds of anonymous loci from across the whole genome and could be used for both population genetic, phylogenetic, and species delimitation studies. It is suited for both well-preserved DNA from silica-gel dried leaf material and degraded DNA from herbarium specimens.

The present AFLPseq study comprised 12 *Adonis* accessions (Supplementary Table S1), either recently collected, silica-gel dried material (five accessions from Italy and Romania), or well-preserved herbarium material housed in the herbaria B and BRNO (seven accessions from Romania, the Russian Federation, and Kazakhstan). The accessions were selected (a) to cover large parts of the distribution range of *A. volgensis* and (b) to include only plant material in the fingerprinting procedure, for which extracts of unfragmented genomic DNA was expectable. The AFLPseq procedure followed the protocol given in [33] with the following modifications: in the restriction-ligation step, we used a double-digestion procedure with restriction enzymes MseI and EcoRI. After ligation of MseI and EcoRI adapters (MseI adapter: 5′-GACGATGAGTCCTGAG-3′ + 5′-TACTCAGGACTCAT-3′; EcoRI adapter: 5′-CTCGTAGACTGCGTACC-3′ + 5′-AATTGGTACGCAGTCTAC-3′), we continued with the AFLP genome-reduction protocol using primers with 1bp-overhangs (MseI-C: 5′-GATGAGTCCTGAGTAAC-3′; EcoRI-A: 5′-GACTGCGTACCAATTCA) in the pre-selective amplification step and in the selective amplification step with additional 1bp- (EcoRI side) or 2bp-overhangs (MseI side), respectively. The two primers used in the latter amplification step, however, were additionally tailored to include Nanopore barcode adapter sequences at the 5′ end of the primers (Mse_CTG_Nanopore_fw: 5′-TTTCTGTTGGTGCTGATATTGCGATGAGTCCTGAGTAACTG-3′; Eco_AA_Nanopore_rv: 5′-ACTTGCCTGTCGCTCTATCTTCGACTCCGTACCAATTCAA-3′), as suggested in the ′Ligation sequencing amplicons—PCR barcoding (SQK-LSK109 with EXP-PBC001)’ protocol by Oxford Nanopore Technologies, substituting a subsequent ligation of the Nanopore barcode adapter with an additional barcoding PCR. To ensure specific binding with long and tailed primers, a two-step variation of the selective PCR was conducted (94 °C for 2 min; followed by 30 cycles of 94 °C for 20 s and 72 °C for 2 min; and a final step at 72 °C for 2 min). To every 2 µL of 1:10 diluted preselective PCR product, 5 µL Taq DNA Polymerase Master Mix RED, 0.25 µL of each 10 µM tailed selective primer, and 2.5 µL H2O were added. After the selective PCR, the length of the fragments ranged from 200–500 bp. All subsequent steps (Nanopore barcode PCR, sample multiplexing, size selection, preparation of Nanopore sequencing library) followed [33]. The resulting library was sequenced with the MinION using a Flongle flow cell. Read data processing, de novo locus assembly, identification of orthologous loci, and reference-based SNP calling with the SLANG pipeline, and the final calculation of frequency-sensitive SNP-based Nei distances followed the protocol described by [33]. Based on these pairwise distances both a phylogenetic network reconstruction using the Neighbor-joining method in SPLITSTREE v4.16.1 [35] and a principal co-ordinate analysis (PCoA) with a custom R v4.0.5 script using the ‘phangorn‘ library to read the distance matrices and the ‘ape‘ package to calculate and plot the PCoA was carried out.

## 3. Results

### 3.1. Morphometric Analyses

**Figure 1:**
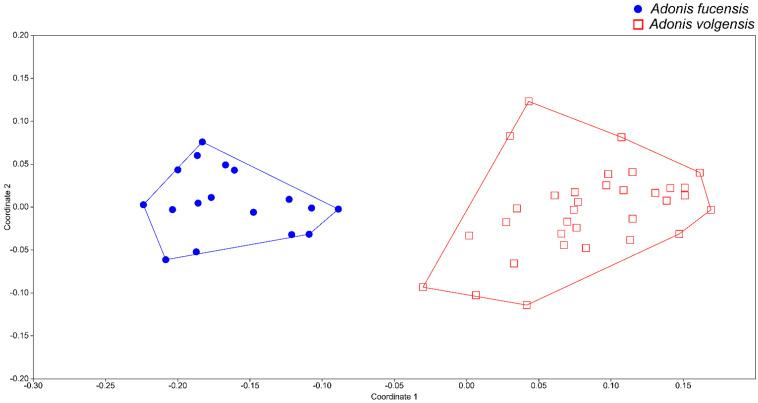
Non-metric multidimensional scaling scatterplot showing the first two dimensions of the analysis.

**Figure 2:**
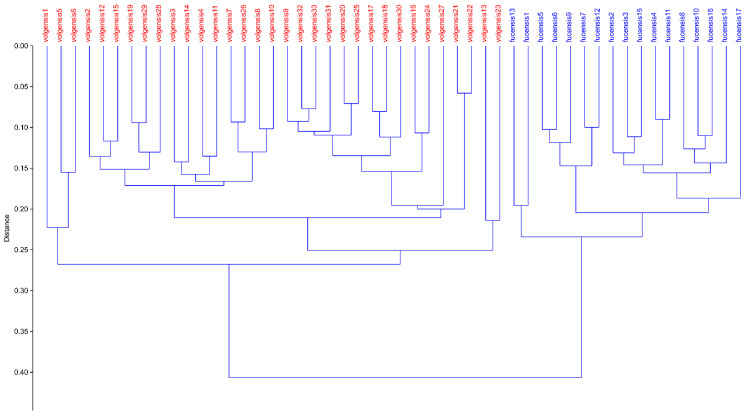
Hierarchical clustering of individuals of *A. volgensis* and *A. fucensis* using paired group algorithm (UPGMA) and Gower Similarity Index. Cophenetic correlation coefficient is 0.8566.

The NMDS, performed with three dimensions, yielded an ordination with a stress value of 0.09224. The scatterplot shows on the first two axes a clear distinction between *A. volgensis* and *A. fucensis*, and no overlapping areas among individuals were found (Figure 1). The UPGMA dendrogram (Figure 2) yielded two well-defined clusters, one including all individuals of *A. volgensis* and the other all individuals of *A. fucensis*.

**Figure 3:**
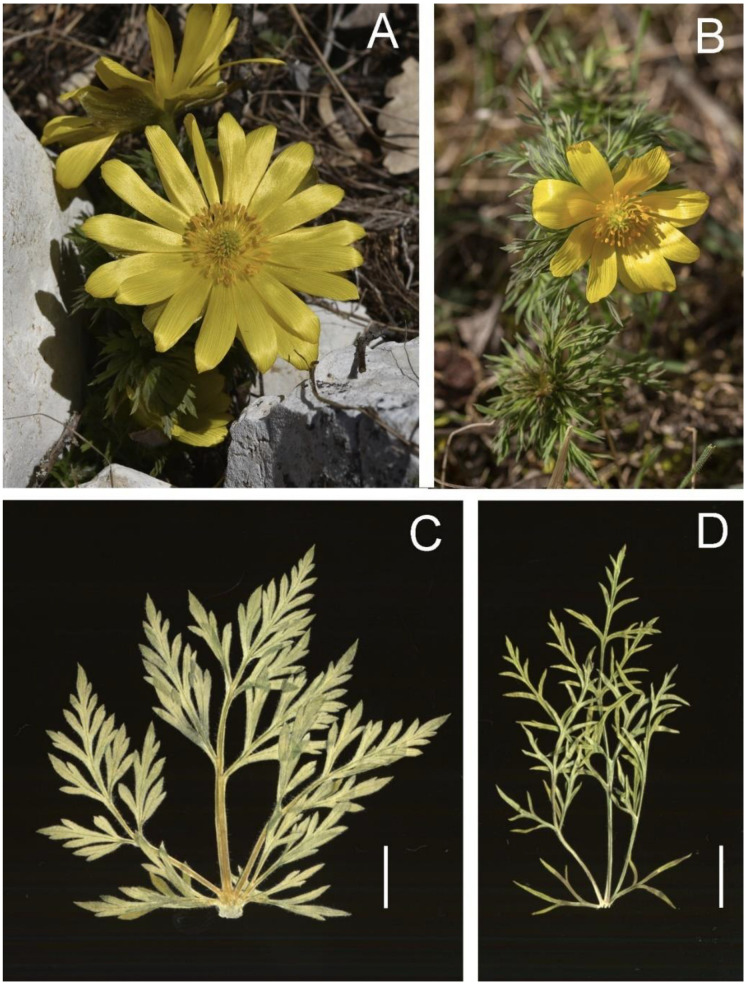
Comparison of *A. fucensis* and *A. volgensis*: (**A**) *A. fucensis* from Mt. Annamunna locality (Italy, Abruzzo, photo by F. Bartolucci); (**B**) *A. volgensis* from Murfatlar locality (Romania, Constanța County, photo by R. Nicoară); (**C**) dry cauline leaf of *A. fucensis*, pentagonal leaf blade; (**D**) dry cauline leaf of *A. volgensis*, triangular-ovate leaf blade. Scale bar 1 cm.

**Figure 4:**
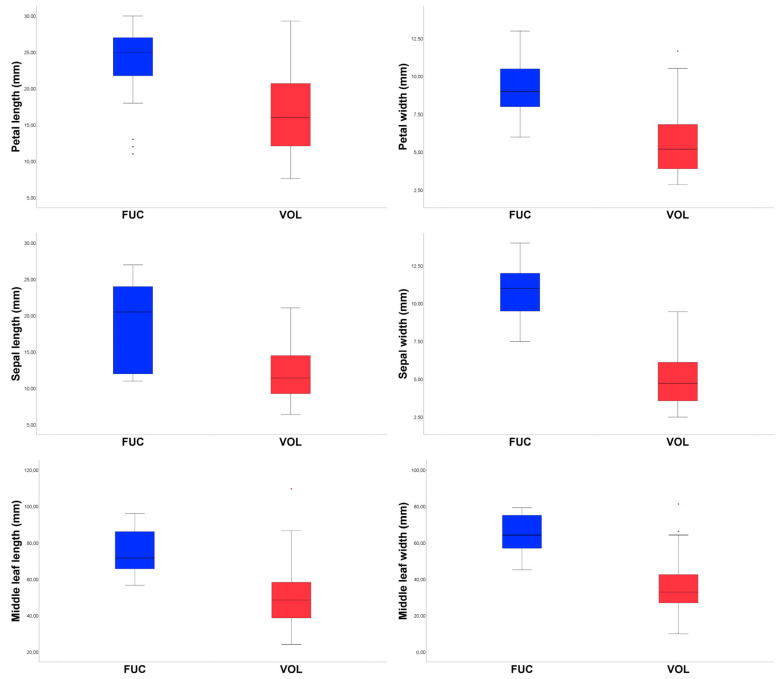

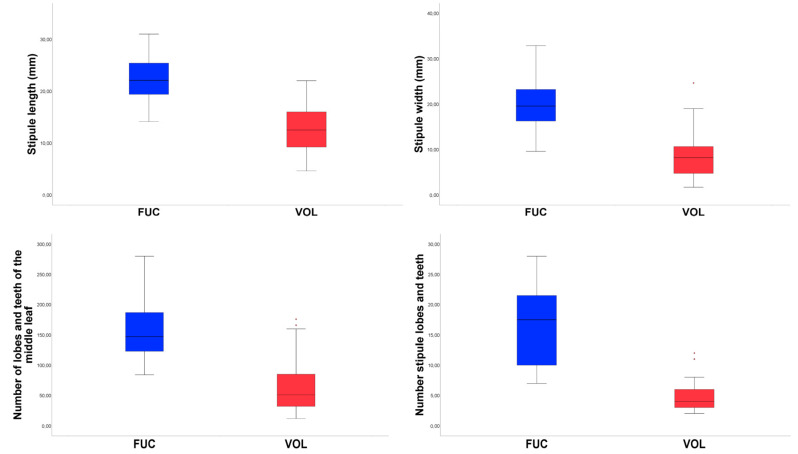
Boxplots expressing morphological variation between *A. fucensis* (FUC) and *A. volgensis* (VOL). Outlined central box depicts middle 50% of data, extending from 25th and 75th percentiles, and horizontal bar is the median. Ends of vertical lines (or “whiskers”) indicate minimum and maximum data values, unless outliers are present, in which case whiskers extend to a maximum of 1.5 times inter-quartile range. Circles indicate outliers.

Comparisons of morphological characters between *A. volgensis* and *A. fucensis* (Figure 3) are summarized in Table 2. The states of 13 characters (H, MLL, MLW, NMLN, ATL, LMW, LWB, LMW/ LWB, SL, SW, NSL, CD, SLD, SWD, PLD, and PWD) show significant differences between the two species (*p* < 0.01). Boxplots of relevant characters are showed in Figure 4.

### 3.2. nrDNA Sequence Variation

**Figure 5:**
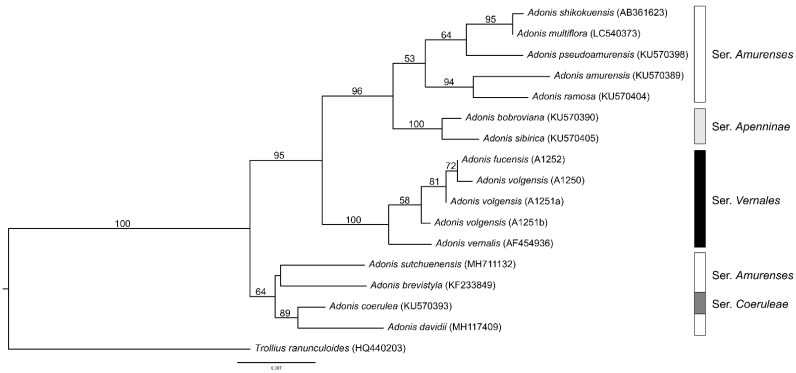
Neighbor-joining tree of 15 accessions of *Adonis* sect. *Adonanthe* based on nrDNA ITS sequence variation and Kimura-2-parameter distances. GenBank accession numbers and probe numbers of the present study (*A. fucensis*, *A. volgensis*) are given in brackets. Numbers above branches are bootstrap values based on 1000 replicates.

The Neighbor-joining tree based on Kimura-2-parameter distances among nrDNA ITS sequences of 15 *Adonis* accessions is shown in Figure 5. The central Italian *Adonis fucensis* accession (A1252) is found being closely related with *A. volgensis* and *A. vernalis* in the monophyletic group of *A.* sect. *Adonanthe* ser. *Vernales*. As also found by a more comprehensive phylogenetic analysis of section *Adonanthe* performed by [4], series *Amurenses* did not form a monophyletic group.

### 3.3. AFLPseq Fingerprinting

**Figure 6:**
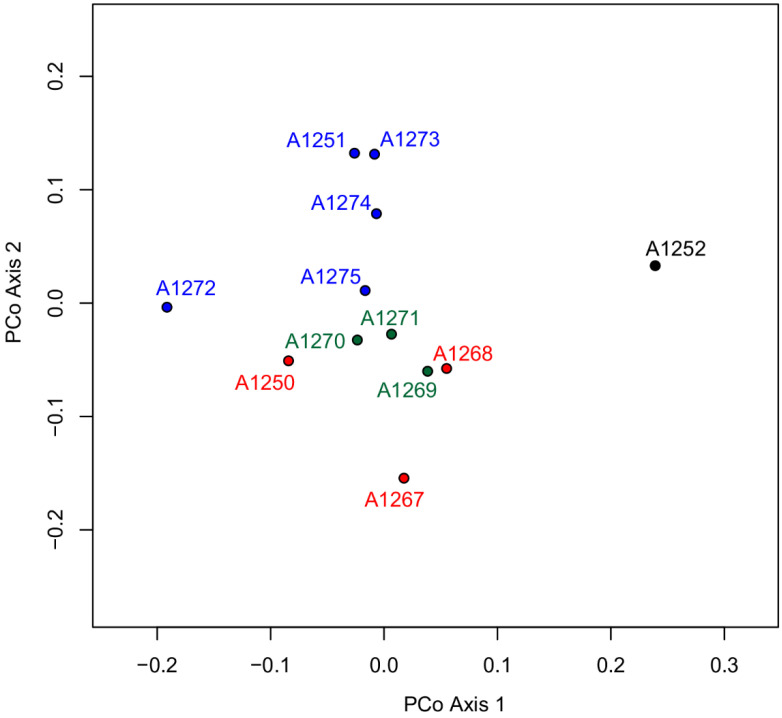
Ordination of accessions of *A. fucensis* (black) and *A. volgensis* (blue: Romania; red: Russia; green: Kazakhstan) based on pairwise Nei distances from 2944 single-nucleotide polymorphisms (SNPs) from 486 AFLPseq loci in a Principal Co-ordinate Analysis (PCoA), with axis 1 explaining 20.8% and axis 2 explaining 15.0% of the total variance, respectively.

In total, 731,698 reads and 243.72 Mbp were sequenced for the 12 *Adonis* accessions. After read preprocessing, 592,432 reads with lengths between 10 bp and 614 bp passed the Q5 quality filter. With the SLANG pipeline (cluster thresholds optimized to values of 0.85 and 0.95 for the first and second cluster step, respectively), 486 orthologous loci were inferred, containing 2944 SNPs. After calculation of pairwise Nei distances, the resulting Neighbor-joining tree (Supplementary File S2) and the PCoA plot were received (Figure 6). While in the first, the *Adonis* accession from the Central Apennines (A1252) is connected with the remaining *A. volgensis* representatives without any exceptionally longer branch than the other accessions, the PCoA plot demonstrates the clear separation between the two taxa; with accessions of the latter on the left and accession A1252 on the right side of principal co-ordinate PCo axis 1, which account for 20.8% of the total variation in the data set. Additionally, PCo axis 2 (accounting for additional 15.0% of the total variation) shows a clear geographical separation within *A. volgensis*, with accessions of this species from Romania (sometimes considered as being an independent species, *A. transsilvanica*) on the positive and accessions from Russia and Kazakhstan on the negative side of the axis.

An additional result of the analysis is worth mentioning in methodological respects: accessions A1251, A1273, and A1274 are very similar to each other in spite of the fact that the three probes come from the same locality (Romania, Constanta, Cotu Văii), but were recently collected as silica-gel dried leaf material (the latter two) or as an herbarium specimen (A1251) twenty years ago. This observation adds to the trustworthiness of the AFLPseq protocol and the comparability of differently preserved DNAs in terms of sequence information retrieved through this process.

## 4. Discussion

Morphological and molecular analyses provide evidence that *A. fucensis* should be regarded as a new species, endemic to Abruzzo (Central Apennines, Italy). It is similar to *A. volgensis*, a typical plant of the E-European and Asiatic steppes, distributed in Hungary, Romania, Bulgaria, and Turkey, as well as eastward to SW Siberia and Central Asia, but it can be distinguished by several quantitative and qualitative morphological characters, as shown in Table 2. The new species lives in shrub-steppe habitat in contact environments between bushes dominated by *Prunus spinosa* L. subsp. *spinosa* and steppe grasslands with the presence of *Festuca valesiaca* Schleich. ex Gaudin subsp. *valesiaca*. Abruzzo is the Italian administrative region with the highest number of taxa belonging to the genus *Adonis*, and also hosts the only Italian populations of the extremely rare steppe species *A. vernalis*.

The dry sub-continental climate of internal basins of the Central Italy, together with wild herbivore disturbance and prehistoric anthropogenic fires [36], may have reduced the post-glacial reforestation. Subsequently sheep grazing and the practice of transhumance, dating back to the 6th century BC or earlier in Abruzzo and widely practiced until the 1950s [37,38], has probably favored the spread of grasslands [39]. Around Amplero, close to the locality of *A. fucensis*, lies an archaeological site inhabited since the VI century B.C. The area hosted, from the Bronze age until Medieval times and beyond, important shepherd settlements and was located on transhumance routes [40]. These causes explain the persistence of steppe species in the internal areas of the Central Apennines.

In the internal basins of Abruzzo such as the Fucino and the L’Aquila plains, there is a consistent number of grassland taxa featuring a disjunction with E-European steppes, giving these areas a pronounced steppic character: *Alyssum desertorum* Stapf., *Androsace maxima* L., *Astragalus danicus* Retz., *A. exscapus* L. subsp. *exscapus*, *A. onobrychis* L., *A. vesicarius* L. subsp. *vesicarius*, *Ceratocephala falcata* (L.) Cramer, *Crocus variegatus* Hoppe & Hornsch, *Falcaria vulgaris* Bernh., *Festuca valesiaca* Schleich. ex Gaudin subsp. *valesiaca*, *Pulsatilla montana* (Hoppe) Rchb. subsp. *montana*, *Poa perconcinna* J.R.Edm., *Salvia aethiopis* L., and *Stipa capillata* L. [12,15,16]. In addition, some Italian endemics living in the same area should be considered of steppic origin such as *Goniolimon tataricum* (L.) Boiss. subsp. *italicum* (Tammaro, Pignatti & Frizzi) Buzurović [41,42], and *Astragalus aquilanus* Anzal. [43]. The presence of these plants in the central Apennines is due to different migrations from east to west. Plants with similar morphological features, with respect to the Northern and Eastern populations, e.g., *A. exscapus* subsp. *exscapus*, or *F. valesiaca* subsp. *valesiaca*, probably arrived in the Central Apennines during late-Pleistocene [15]. An initial wave of steppic plants probably occurred during the Messinian Salinity Crisis, such as the spread of an ancient *G. tataricum* lineage throughout south-eastern Europe [37]. This could be a hypothesis on the origin of the presence of *A. fucensis* in the Central Apennines.

Alternatively, a recent study on the phylogeography of the closely related *A. vernalis* [5] revealed that this plant species expanded its range from SE Europe into the Euro-Siberian steppe, with a Spanish population of the species being the earliest-diverging lineage. Whether members of our present study group parallel this migration pattern and *A. fucensis* constitutes the earliest-diverging remnant of an eastwards expanding *A. volgensis* could be hypothesized here but must await a much denser sampling of the latter species. Due to the restricted number of accessions analyzed in the present contribution, the biogeographical history of *A. fucensis* and *A. volgensis* remain unresolved.

*Adonis fucensis* is a very rare species, consisting of a very small population of 65 individuals, assessed here as critically endangered. In the two years (2021–2022) in which we were able to study the population we observed that although plants have a large number of flowers, they produce few fruits (we have observed many abortive achenes), and its survival is probably related to vegetative reproduction with consequent loss of genetic diversity. It will be absolutely necessary to undertake a dialogue with the National Park of Abruzzo, Lazio, and Molise to plan correct in situ and ex situ conservation strategies, to try to save this new species from extinction.

## 5. Taxonomic Treatment

***Adonis*** subg. ***Adonanthe*** (Spach) W.T.Wang, Bull. Bot. Res., Harbin 14(1): 22 (1994) ≡ *Adonanthe* Spach, Hist. Nat. Vég. 7: 227 (1838).

Type: *A. vernalis* L. [Lectotype, Herb. Linn. No. 714.4, LINN [digital photo!]); image of the lectotype is available at https://linnean-online.org (accessed on 28 November 2022)].

***Adonis*** sect. ***Adonanthe*** W.T.Wang, Bull. Bot. Res., Harbin 14(1): 26 (1994).

Type: A. vernalis L.

***Adonis*** ser. ***Vernales*** Bobr. ex Poschk., Novosti Sist. Vyssh. Rast. 14: 83 (1977).

Type: A. vernalis L.

***Adonis volgensis*** DC., Syst. Nat. [Candolle] 1: 545 (1817) ≡ *Adonanthe volgensis* (DC.) Chrtek & Slavíková, Preslia 50(1): 24 (1978).

Holotype: *Ad Volgam*, 1817, *Steven s.n.* [G barcode G00144834 [digital photo!]; image of the holotype is available at http://www.ville-ge.ch/musinfo/bd/cjb/chg (accessed on 28 November 2022)].

= *Adonis transsilvanica* Simonovich, Dokl. Akad. Nauk Belorusski. S.S.R., IX(6): 396 (1965).

Holotype: Hungaria. Transsilvania. *In collibus herbidis ad “Szénafü” prope “Kolozsvár”*, May and June 1910, *A. Richter 5201* [LE barcode LE00012366 [digital photo!], isotypes LD No. 2196730 [digital photo!], S No. 7488 [digital photo!]; image of the holotype is available at https://en.herbariumle.ru (accessed on 28 November 2022)].

**Figure 7:**
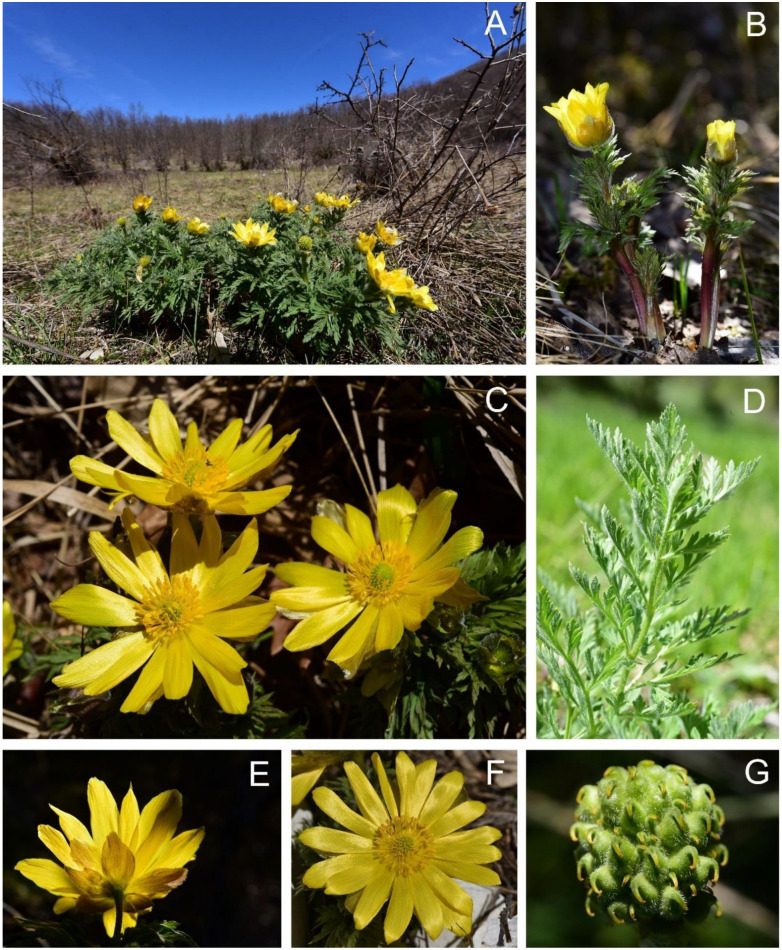
*Adonis fucensis* F.Conti & Bartolucci [Italy, Abruzzo, Mt. Annamunna, photo by F. Conti (**A**–**E**,**G**) and (**F**). Bartolucci (F)]. (**A**) Habitat and flowering plants of *A. fucensis*; (**B**) whole plants; (**C**) flowering plants; (**D**) cauline leaf; (**E**) flower, dorsal view; (**F**) flower, front view; (**G**) not mature aggregate fruit.

***Adonis fucensis*** F.Conti & Bartolucci, sp. *nov.* (Figure 7).

Type: ITALY. Abruzzo, Valle Lupara alla base del Monte Annamunna (Collelongo, L’Aquila; WGS84 33T 41°55′21″ N, 13°38′8″ E), radura, margini e cespuglieti a *Prunus spinosa* L. subsp. *spinosa*, 1038 m, 9 April 2021, *F. Conti s.n.* (holotype APP No. 66211).

Diagnosis: it is similar to *A. volgensis* but can be distinguished by larger cauline leaves, leaf blade pentagonal vs. triangular-ovate, lobes lanceolate vs. linear to narrowly lanceolate, larger stipules more divided, and larger sepals and petals.

Description: Herbs perennial. Rhizomatous. Roots numerous, fibrous. Stems erect, branched, pubescent, (8)10.25–13.75(16) cm tall. Scales membranous, alternate at lower parts of stem. Leaves stipulate, alternate, palmately compound with segments 3-pinnatifid, sessile, pentagonal, (56.7)65.75–86.22(96.06) mm long, (45.16)57.01–75.06(79.30) mm wide, green, hairy, n. of lobes and teeth (84)123–187(280), terminal lobes lanceolate, dentate, mucronate to acute at apex with angle (31.73)43.36–54.32(74.4)°, subterminal lobes (3.3)3.5–5.7(6.7) mm long, (1.14)1.41–1.91(2.83) mm max wide, (0.90)1.08–1.49(2.29) mm wide at base; stipules pinnatifid, (14.13)19.83–25.23(31.00) mm long, (9.59)16.40–23.24(32.87) mm wide, n. of stipules lobes and teeth (7)10–21(–28). Flower solitary, (40)44.75–60(66) mm in diameter in vivo, yellow. Sepals 5–8, ovate, obovate to elliptic, rarely truncate or dentate, (17)19–27.8(33) mm long (fresh), (11)12.25–23.63(27) mm long (dry), (9)10.1–14.9(18) mm wide (fresh), (7.5)9.63–12(14) mm wide (dry), olive-green, yellowish, brownish, purplish abaxially, hairy. Petals (9)12–14(18), yellow, obovate to narrowly obovate, (19)21–32(33) mm long (fresh), (11)21.75–27(30) mm long (dry), (7–)8–12.9(–14) mm wide (fresh), (6)8–10.50(13) mm wide (dry), obtuse, entire or rarely with rounded notches at apex, glabrous. Stamens numerous, basifixed; anther 2-loculed, oblong, 2–2.2 mm long, 0.5–0.7 mm wide; filaments filiform ca. 6 mm. Pistil numerous, ovary ovate, puberulous; styles 0.9–1.5 mm long, recurved. Aggregated fruit subglobular to ellipsoidal, 9–11 mm long, 8–10 mm wide. Receptacle elliptical pubescent. Achenes numerous, obovoid to ellipsoidal, 3.5–4.3 mm long, 2.5–3.2 mm wide, pubescent; style 0.9–1.5 mm long, recurved at base, ± appressed.

Etymology: *Adonis fucensis* is named after Fucino Plain located nearby to the north and affected by the presence of the third largest Italian lake drained in 1878.

Habitat: The species grows in the contact zone between bushes dominated by *Prunus spinosa* subsp. *spinosa* and steppic grasslands with *Festuca valesiaca* subsp. *valesiaca*, *Achillea setacea* Waldst. & Kit., *Koeleria splendens* C.Presl, *Centaurea jacea* L. subsp. *gaudinii* (Boiss. & Reut.) Gremli, and *Galium verum* L. subsp. *verum*.

Phenology: Flowering from March to April; fruiting from May to June.

Distribution: Endemic to one locality of Abruzzo (Central Italy) within the SAC IT7110205 “Parco Nazionale d’Abruzzo”. The species grows in a small flat clearing on the slopes of Mt. Annamunna, between Amplero and Fucino plains (Supplementary File S1).

Conservation status: *Adonis fucensis* is known only by one location (locus classicus) where, during 2021, we counted only 65 individuals (genets). It is located within the NATURA 2000 network in the SAC IT7110205 “Parco Nazionale d’Abruzzo”. The area of occupancy (AOO) is 4 km^2^ (cell grid 2 × 2 km), calculated with GeoCAT (Geospatial Conservation Assessment Tool) software (http://www.plantsoftheworldonline.org/ (accessed on 10 October 2022)) [44]. We observed pressure due to the grazing of wild animals (especially wild boars that dig up single plants). Observing the aerial photos of the 1980s it is evident how in the *A. fucensis* habitat the shrub and tree vegetation increased by reducing the surface of the pastures probably due to a decrease in grazing by livestock. The natural succession of vegetation is a pressure and a threat for the population of *A. fucensis*. It is not possible to be certain of the decline of the species even if it is reasonable to assume that it was more common in the past. According to IUCN criterion B2ab(iii) [45], the species is assessed as Critically Endangered (CR).

### Key to Adonis Species Belonging to sect. Adonanthe Distributed in Europe

1. Leaves pinnately compound …………………………………………………... ***A. apennina***
1. Leaves palmately compound …………………………………………………….………… 2
2. Leaves glabrous, with narrowly linear, entire lobes, rarely few-dentate ........ ***A. vernalis***
2. Leaves pubescent, rarely glabrescent, with linear-lanceolate to lanceolate, dentate lobes …………………………………………………………………………………………………….. 3
3. Middle cauline leaves triangular-ovate, rarely pentagonal, (24.12)38.70–58.35(109.66) mm long × (10.00)27.00–42.60(81.30) mm wide, with (12)32–85(176) lobes and teeth linear to narrowly lanceolate; stipule (4.64)9.31–15.88(22.00) mm long, with (2–)3–6(–12) lobes and teeth; sepal (6.40)9.26–14.51(21.07) mm long × (2.50)3.57–6.05(9.46) mm wide, petal (7.61)12.12–20.69(29.28) mm long × (2.85)3.95–6.83(11.68) mm wide ………………….………………………………………………………..………..…. ***A. volgensis***
3. Middle cauline leaves pentagonal, (56.70)65.75–86.22(96.06) mm long × (45.16)57.01–75.06(79.30) mm wide, with (84)123–187(280) lobes and teeth lanceolate; stipule (14.13)19.83–25.23(31.00) with 10–22.4(–26) lobes and teeth; sepal (11.00)12.25–23.63(27.00) mm long × (7.50)9.63–12.00(14.00) mm wide, petal (11.00)21.75–27.00(30.00) mm long × (6.00)8.00–10.50(13.00) mm wide …………………………………………………… ***A. fucensis***

## Figures and Tables

**Table 1:** Morphological characters studied.

Abbreviation	Description of the Character	Type
H	Height (cm)	quantitative continuous
MLL	Middle leaf length (mm)	quantitative continuous
MLW	Middle leaf width (mm)	quantitative continuous
SML	Shape of middle leaf (0: pentagonal; 1: triangular-ovate)	qualitative
NLML	Number of lobes and teeth of the middle leaf	quantitative discrete
STL	Shape of terminal lobe (0: lanceolate; 1: narrowly lanceolate; 2: linear)	qualitative
ATL	Angle of terminal apex lobe (°)	quantitative continuous
LMW	Subterminal lobe max width (mm)	quantitative continuous
LWB	Subterminal lobe width at base (mm)	quantitative continuous
LMW/LWB	Ratio lobe max width/lobe width at base	calculated ratio
SL	Stipule length (mm)	quantitative continuous
SW	Stipule width (mm)	quantitative continuous
NSL	Number stipule lobes and teeth	quantitative discrete
SLD	Sepal length (mm)	quantitative continuous
SWD	Sepal width (mm)	quantitative continuous
PLD	Petal length (mm)	quantitative continuous
PWD	Petal width (mm)	quantitative continuous
NP	Number of petals	quantitative discrete

**Table 2:** Morphological comparison of *A. volgensis* and *A. fucensis*. Quantitative continuous characters are expressed in mm and are reported as mean ± standard deviation and 25–75 percentiles (extreme values in brackets). For quantitative discrete cardinal characters, 25–75 percentiles are given (extreme values in brackets). Significantly different character states are shown in bold (*p* < 0.01).

	*Adonis volgensis*		*Adonis fucensis*	
Height (cm)	**22.80 ± 7.21**	**(10.49)17.42–27.39(39.61)**	**12.06 ± 2.46**	**(8)10.25–13.75(16)**
Middle leaf length (mm)	**50.36 ± 16.86**	**(24.12)38.70–58.35(109.66)**	**74.28 ± 12.39**	**(56.70)65.75–86.22(96.06)**
Middle leaf width (mm)	**35.01 ± 13.75**	**(10.00)27.00–42.60(81.30)**	**64.31 ± 10.83**	**(45.16)57.01–75.06(79.30)**
Shape of middle leaf		triangular-ovate, rarely pentagonal		pentagonal
Number of lobes and teeth of the middle leaf		**(12)32–85(176)**		**(84)123–187(280)**
Shape of terminal lobe		linear to narrowly lanceolate		lanceolate
Angle of terminal apex lobe (°)	**31.95 ± 11.21**	**(15.04)23.73–37.55(64.96)**	**50.45 ± 11.76**	**(31.73)43.36–54.32(74.4)**
Subterminal lobe max width (mm)	**1.13 ± 0.39**	**(0.53)0.84–1.34(2.39)**	**1.76 ± 0.48**	**(1.14)1.41–1.91(2.83)**
Subterminal lobe width at base (mm)	**0.94 ± 0.28**	**(0.51)0.70–1.10(1.75)**	**1.36 ± 0.38**	**(0.90)1.08–1.49(2.29)**
Ratio lobe max width/lobe width at base	1.21 ± 0.27	(0.91)1.02–1.32(2.52)	1.29 ± 0.11	(1.13)1.21–1.37(1.57)
Stipule length (mm)	**12.63 ± 4.36**	**(4.64)9.31–15.88(22.00)**	**22.41 ± 4.53**	**(14.13)19.83–25.23(31.00)**
Stipule width (mm)	**8.45 ± 4.82**	**(1.69)4.85–10.60(24.63)**	**22.06 ± 5.56**	**(9.59)16.40–23.24(32.87)**
Number of stipule lobes and teeth		**(2–)3–6(–12)**		**(7)10–21(–28)**
Sepal length (mm)	**12.23 ± 3.67**	**(6.40)9.26–14.51(21.07)**	**18.81 ± 5.80**	**(11.00)12.25–23.63(27.00)**
Sepal width (mm)	**4.98 ± 1.71**	**(2.50)3.57–6.05(9.46)**	**10.61 ± 1.82**	**(7.50)9.63–12.00(14.00)**
Petal length (mm)	**17.2 ± 6.04**	**(7.61)12.12–20.69(29.28)**	**23.48 ± 5.54**	**(11.00)21.75–27.00(30.00)**
Petal width (mm)	**5.59 ± 2.15**	**(2.85)3.95–6.83(11.68)**	**9.37 ± 1.96**	**(6.00)8.00–10.50(13.00)**
Number of petals		(10)12–13(16)		(9)12–14(18)

## Data Availability

The data presented in the current study are available within the article and Supplementary Materials.

## Supplementary Materials

The following supporting information can be downloaded at: https://www.mdpi.com/article/10.3390/biology12010118/s1, File S1: List of the herbarium specimens examined; distribution map of *Adonis fucensis*. File S2: Neighbor-joining tree of accessions of *Adonis fucensis* and *A. volgensis*. Table S1: *Adonis* populations sampled for the present study with information on localities, voucher specimens, and GenBank accession numbers for nrDNA ITS.
