# Supplementary material for: Adonis fucensis (A. sect. Adonanthe, Ranunculaceae), a New Species from the Central Apennines (Italy)
Source: Biology (Basel). 2023 Jan 11;12(1):118. doi: 10.3390/biology12010118 (PMC9855803; doi:10.3390/biology12010118)
Supplement: Supplementary file 1 [file biology-12-00118-s001.zip › Sup_fileS1.pdf]

Article: *Adonis fucensis* (*A.* sect. *Adonanthe*, Ranunculaceae), a new species from the Central Apennines (Italy)

Authors: Fabio Conti, Christoph Oberprieler, Marco Dorfner, Erik Schabel, Roxana Nicoară, Fabrizio Bartolucci

Supplementary File S1.

List of the specimens examined

Adonis volgensis DC.

Romania. Fântânița Murfatlar Natural Reserve, Murfatlar locality, Constanța County (44.15936° N, 28.39118° E), 65 m, ponto-sarmatic steppes with xerophytic vegetation with *Chrysopogon gryllus, Festuca valesiaca, Amygdalus nana, Euphorbia nicaeensis, Eryngium campestre*, 7 April 2022, *Nicoară R. s.n.* (APP No. 66217); Fânațele Clujului Natural Reserve, Cluj county (46.815361° N 23.659750° E), 471 m, *Crategus monogyna* scrub with *Poa angustifolia, Veronica chamaedrys, Fragaria vesca, Galium* sp., *Euphorbia cyparissias, Plantago media, Glechoma hirsuta*, 7 May 2022, *Nicoară R. s.n.* (APP No. 66215); Cotu Văii locality, Constanța County (43.808369°N 28.335304°E), 75 m, steppes with xerophytic vegetation with *Ajuga chamaepytis, Achillea clypeolata, Cerastium* sp., *Salvia nutans, Paeonia tenuifolia*, 1 May 2022, *Nicoară R. s.n.* (APP No. 66214); Cotu Văii locality, Constanța County, (43.829096° N 28.353753° E), 61 m, grazed steppes with xerophytic vegetation with *Paliurus spina-christi, Thlaspi arvense, Marrubium vulgare, Adonis flammea, Plantago lanceolata, Artemisia austriaca*, 1 May 2022, *Nicoară R. s.n.* (APP No. 66216); Comit. Békés. *Ad aggerem viae ferreae prope Csorvás, versus Orosháza*, 97 m, 20 May 1938, *A. Boros s.n.* (BP barcode HNHM-TRA 00035292; UPS No. V-946907); Békés m., Csorvás, a vzsnti [?] toldes [?] mentén Orosháza felé, pusztnloban [?], 6 April 1948, *S. Jávorka s.n.* (BP barcode HNHM-TRA 00035296); in collibus Klausenburg, 3-5 April 1877, *J. Barth s.n.* (BP barcode HNHM-TRA 00035285); in campestribus elatioribus Szénafü prope Klausenburg Transilvaniae, 29 May 1868, *Janka s.n.* (BP barcode HNHM-TRA 00035281); ibidem, 7 April 1870, *Janka s.n.* (BP barcode HNHM-TRA 00035284); Clausenburg hynn minfnu [?], Elövölgy, March April 1856, zun… [?] vrufinfunchen [?], *J. Wolff s.n.* (BP barcode HNHM-TRA 00035287); Csorvás, Orosházi Lanjáh [?] … [?], *Herkner s.n.* (BP barcodes HNHM-TRA 00035290, 00035291); in foenatis Elövölgy ad oppidum Claudiopolis (Kolozsvár), 450 m, 10 May 1940, *E.I et A. Nyárády s.n.* (BP barcode HNHM-TRA 00035258); Claudiopoli, in herbidis, solo argill. 400 m s.l.m., April 1887, *J. Wolff s.n.* (BP barcode HNHM-TRA 00035260; CL No. 72730); Siebenbogen. Comitat: Kolos, Bergwiesen bei Klausenburg, Tert. Tegel 460 m, 18 April 1872, *J. Freyn s.n.* (BP barcode HNHM-TRA 00035267); Hungaria, Transsilvania, in collibus herbidis ad Szénafü prope Kolozsvár, May-June 1910, *A. Richter s.n.* (BP barcode HNHM-TRA 00035275); ibidem, 3 April 1911 (fl.), 26 April 1911 (fol.) *A. Richter s.n.* (BP barcode HNHM-TRA 00035274); Kolosvár: Szénafü, 15 May 1907, *A. Richter s.n.* (CL No. 13579); in reg. oppid. Kolosvár: in prat. mont. Szénafü, 15 April 1905, *A. Richter s.n.* (US No. 3575883); Gád Vüsoara r. Colmatui reg. Iolati, 23 April 1953, *I. Suba*… [?] *s.n.* (CL Nos. 509878, 580684); Transsilvania, distr. Cluj, Fînatele Clujului, 17 April 1991, *G. Groza s.n.* (CL No. 653198); Distr. Constanța, in declivibus calcareis ad Fîntînița prope vicum Murfatlar, alt. ca. 50 m, 3 April 1970, *G. Sălăgeanu et C. Horeanu s.n.* (CL No. 601000); Distr.

Constanţa, Cotul Văii, Valea Mare, in herbosis (loess), 43°48′29″N 28°20′08″E, 85 m, 27 April 2002, *G. Negrean s.n.* (CL No. 660517); Distr. Constanţa, Cotul Văii S, Valea Cotul Văii, La lc., platou, in herbosis, 43°48′27,81″N 28°20′06,84″E, 73 m, 27 April 2002, *G. Negrean s.n.* (CL No. 667807); Muntenia, distr. Ilfov., ad silvas, Fantána cu nuc penes Comana, 10 May 1927, *A. Borza s.n.* (CL No. 500870); Cluj: Klausenburg (Cluj Napoca), in pratis, 24 April 1882, *J. Barth s.n.* (UPS No. V-946905); Transylvania centralis, Comitaus Wolozs, e collibus herbidis "Heuwiesen" prope urbem Klausenburg, 3 April (flor.), 4 May (fruct.) 1877, *J. Barth s.n.* (US barcode 03575800); Transilvania (US barcode 03575834); Transsilvania. In collibus herbidis ad "Szénafü" prope "Kolozsvár", May and June 1910, A. Richter 5201 (LE barcode LE00012366 holotype of *A. transsilvanica*, isotypes LD No. 2196730, S No. G-7488; **Moldavia**. Moldavia, distr. Vaslui. In vico dicto "Codăieşti". In loco nominato "Movila lui Burcel", alt. 270 m, 7 April 1967, *E. Ţopa et I. Ostaciuk s.n.* (CL No. 645246); **Russia**. Ad Volgam, 1817, *Steven s.n.* (G barcode G00144834, holotype); Tanaïs, 1837, Herb. Benthamianum (K barcode 000694287); Rossiyskaya Federatsiya, Omskaya oblast', Lyubinskiy rayon, okrestnosti s. Uvalo-Yadrino. Zemlyanichno-kostretsovyy sukhodol'nyy lug., 22 May 2010, *G.V. Samoylova, N.V. Plikina, A.N. Yefremov s.n.* (MW barcode MW0113322); Rasteniya Sredney Rossii, Tul'sk. i Ryazans. gub. na ber. Dona (Yepif. i Dank. uu.), 5 March 2019, *B.D. Vladimirovna 19 a* (MW barcode MW0362120); Plantae prov. Tambow, Usmanskiy u. Bl. d. Kozel'ki. Step', 21 April 1917, *P.A. Smirnov s.n.* (MW barcodes MW0362121, MW0362123, MW0362126, MW0362127, MW0362128); ibidem, 29 April 1917, *P.A. Smirnov s.n.* (MW barcodes MW0362136); Plantae prov. Tambow, Usmanskiy u. Bl. d. Kozel'ki. Step', vostochnyy uchastok, 12 May 1917, *P.A. Smirnov s.n.* (MW barcode MW0362131); Flora prov. Tambow, Step' Leykhtenbergskogo bl. s. Ivanovki Tamb. u., 2 June 1919, *P.A. Smirnov 45* (MW barcode MW0362124); Voronezhskaya obl., Kalacheyevskiy r-n. Dubrava na pravom korennom beregu r. Tolucheyevoy mezhdu sadom sel'khoztekhnikuma i kordonom, 14 May 1946, *N.N. Kaden 325* (MW barcodes MW0362125, MW0362138); … [?], 25 May 1980, *K. Aleksandrova s.n.* (MW barcode MW0362132); Tambovskaya obl., Mordovskiy r-n. V 1 km vostochneye zh.-d. platf. Streletskaya. Lugovina, 14 May 2006, *A. Sukhorukov s.n.* (MW barcode MW0362133); Voronezhskaya obl., Rossiya. 1 km yugo-vostochneye p.g.t. Panino, Paninskiy r-on, opushka osinovogo kusta "Politotdel'skiy"; na chernozemnom grunte, 10 May 1999, *A.V. Slavgorodskiy s.n.* (MW barcode MW0362134); Voronezhskaya obl., Kalacheyevskiy r-n. Les Acer + Ulmus na pravom ber. r. Tolucheyevoy v 6 km k yugu ot sl. Kalach, 6 May 1941, *N.N. Kaden 324* (MW barcode MW0362139); Tambovskoy gub. i u. Step' Leykhtenbergskogo bl. s. Ivanovki, 17 May 1919, *P. Smirnov s.n.* (MW barcode MW0362140); Bashkiriya (b. Ufimsk. i Orenburgsk. g.g.). S. Burangulovo. Lugovaya step', oz. Asli-kul', 24 June 1926, *A.K. Noskov s.n.* (MW barcode MW0362184); Bashkiriya (napr. Ufimsk. i Orenburgsk. i dr.). Step' na sklonakh Balkantau, 2 May 1927, *A.K. Noskov s.n.* (MW barcode MW0362185); s.l., 1877, *A.P. Vial' s.n.* (MW barcode MW0362194); Szossitz comm., … [?] (MW barcode MW0362195); 1875-88. obl. Voysko-Donskogo.: okrest i g. Taganroga i Novocherkasska, *I.F. Kramsakov s.n.* (MW barcode MW0362197); Severnyy sklon. Step' Konskiy Otvod Veshensk. Rayona Donetskogo Okr. Sev.-Kavkaz. Kraya, 5 June 1927, *P. Zhudova s.n.* (MW barcode MW0362198); Donskaya obl. st. Aleksikovo. KH.D.YA. Khut. Dedov Yar. Balka. Sredina lesa. Chasto, s.d., *A. Noskov s.n.* (MW barcode MW0362200); Moldavskaya mest stantsiya pereval. (s. Kornets okr. s. Romanovki. Bel'skaya stat', 11 May

1957, *H. Hrosset s.n.* (MW barcode MW0362201); Russian Federation: Prov. Tambow, Distr. Mordowo, 1 km O von der Eisenbahnstation Streletzkaja, 14 May 2006, *Sukhorukov A. s.n.* (B barcode B 10 0210009); Romania: Cotul Vaii SSW, Valea Mare. in herbosis, 85 m, 27 April 2022, *Negrean G. s.n.* (B barcode B 10 0210277); Russian Federation; Bashkortostan Republic, Baimak distr., Semenovkkoe village (7 km S of Baimak): Tikile-tau hill (634 m), ca 3 km WNW of the village [52°30'59.7'' N 58°17'10.7'' E], 618 m a.s.l., Caragana frutex steppic scrub, 21 July 2007, *M. Kočí, L. Tichý & M. Horsák ex Iter uralense no. 2007/021* (BRNU No. 591605); Russian Federation; Bashkortostan Republic, Abzanovo distr., Verkhnii Mainak village: slopes above the right side of the road to Kugarchi 3.5 km NNW of the village [52°04'10.9'' N 56°44'15.8'' E], 327 m a.s.l., *Stipa* steppe, *M. Chytrý, Z. Otýpková & Z. Lososová ex Iter uralense no. 2007/022* (BRNU No. 591606); Kazakhstan, prov. Karaganda, distr. Abaisk Topar, ca 6 Km E of the village; 624 m a.s.s., meadow steppe with Stipa zalesskii, 49°30'24.5N 72°54'17.5'' E, 30 June 2014, J. *Danihelka, P. Dřevojan & S. Kubešová ex Iter kazachstanicum 2014 no. 786* (BRNU No. 653944); Kazakhstan, prov. Akmola, distr. Ereymentau, Ereymentau, hilly landscape ca. 6 km of the village; 454 m a.l.m., meadow steppe *with Helictotrichon desertorum*, 51°34'30'' N 73°08'17.8'' E, 27 June 2014, *J. Danihelka, P. Dřevojan & S. Kubešová ex Iter kazachstanicum 2014 no. 392* (BRNU No. 653564); Kazakhstan, prov. Akmola, distr. Ereymentau, Ereymentau, hilly landscape ca. 6 km SSE of the village; 446 m a.l.m., , dry steppe, 51°34'17.3'' N 73°08'11.1'' E, 27 June 2014, *M. Chytrý & M. Hájek ex Iter kazachstanicum 2014 no. 393* (BRNU No. 653565).

Adonis fucensis F.Conti & Bartolucci

Italy. Abruzzo, Valle Lupara alla base del Monte Annamunna (Collelongo, L'Aquila; WGS84 41°55′21″ N, 13°38′8″ E), radura, margini e cespuglieti a *Prunus spinosa* L. subsp. *spinosa*, 1038 m, 19 March 2021, *F. Bartolucci and F. Conti s.n.* (holotype APP No. 66208); *ibidem*, 30 March 2021, *F. Bartolucci and F. Conti s.n.* (APP No. 66209); *ibidem*, 9 April 2021, *F. Conti s.n.* (APP No. 66211, Holotypus); *ibidem*, 23 April 2021, *F. Conti and V. Giacanelli s.n.* (APP No. 66210); *ibidem*, 14 June 2021, *F. Bartolucci and F. Conti s.n.* (APP No. 66093).

Distribution map of *Adonis fucensis*.
The map was created using the free and open source QGIS ver. 3.26.2. software [1] and was used under a CC BY-SA copyright from OpenStreetMap contributors.

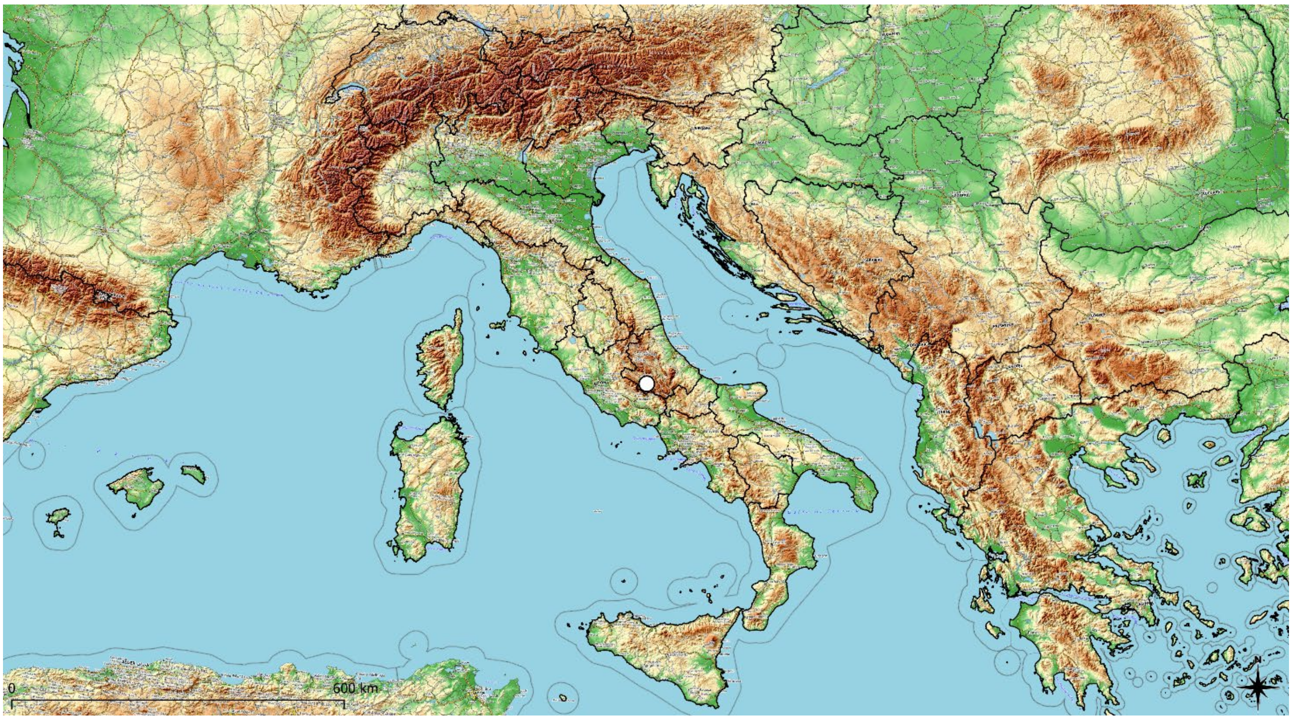

References

1. QGIS. QGIS Geographic Information System. Open Source Geospatial Foundation Project. Availabe online: http://qgis.osgeo.org (accessed on 10 October 2022).
