# Supplementary material for: Adonis fucensis (A. sect. Adonanthe, Ranunculaceae), a New Species from the Central Apennines (Italy)
Source: Biology (Basel). 2023 Jan 11;12(1):118. doi: 10.3390/biology12010118 (PMC9855803; doi:10.3390/biology12010118)

Article: *Adonis fucensis* (*A.* sect. *Adonanthe*, Ranunculaceae), a new species from the Central Apennines (Italy)

Authors: Fabio Conti, Christoph Oberprieler, Marco Dorfner, Erik Schabel, Roxana Nicoară, Fabrizio Bartolucci

Supplementary File S2

Neighbor-Joining tree of accessions of *Adonis fucensis* and *A. volgensis*.

Neighbor-Joining tree of accessions of *Adonis fucensis* (black) and *A. volgensis* (blue: Romania; red: Russia; green: Kazakhstan) based on pair-wise Nei distances from 2,944 single-nucleotide polymorphisms (SNPs) from 486 AFLPseq loci.

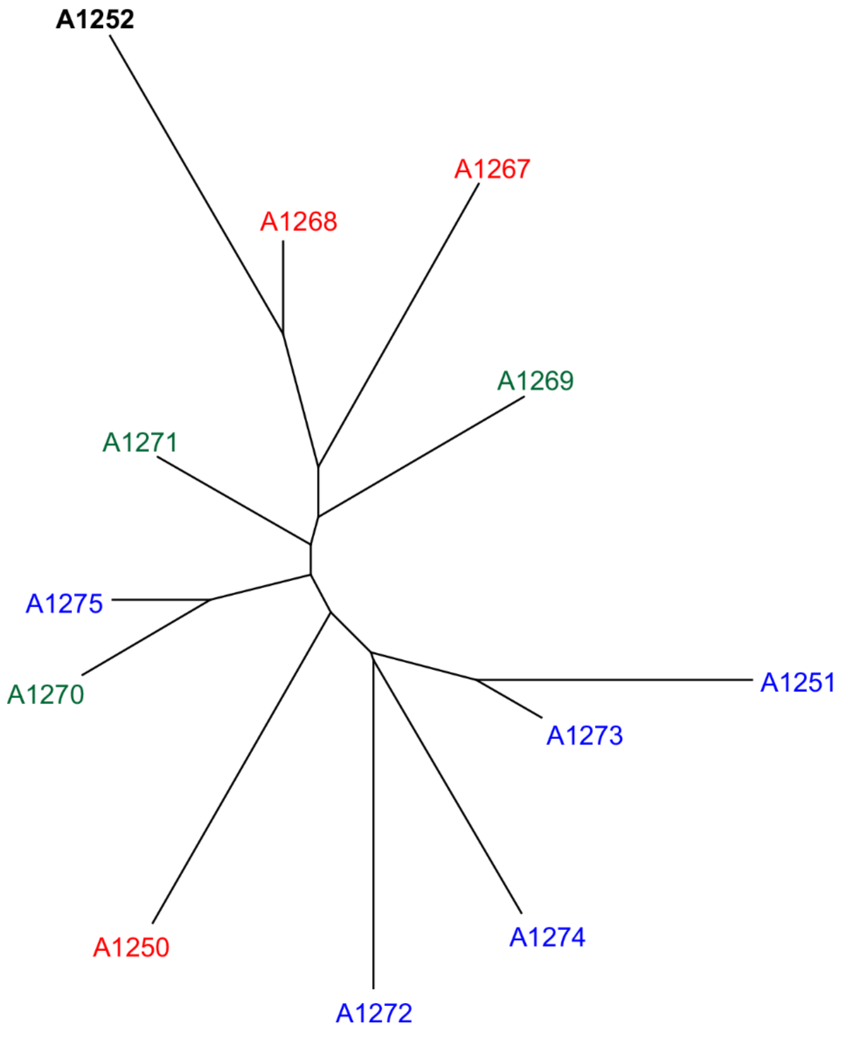

Supplement: Supplementary file 1 [file biology-12-00118-s001.zip › Sup_fileS2.pdf]
