# Supplementary material for: Adonis fucensis (A. sect. Adonanthe, Ranunculaceae), a New Species from the Central Apennines (Italy)
Source: Biology (Basel). 2023 Jan 11;12(1):118. doi: 10.3390/biology12010118 (PMC9855803; doi:10.3390/biology12010118)
Supplement: Supplementary file 1 [file biology-12-00118-s001.zip › Sup_Table-S1.pdf]

Article: *Adonis fucensis* (*A.* sect. *Adonanthe*, Ranunculaceae), a new species from the Central Apennines (Italy)
Authors: Fabio Conti, Christoph Oberprieler, Marco Dorfner, Erik Schabel, Roxana Nicoară, Fabrizio Bartolucci

Supplementary File.

Table S1. *Adonis* populations sampled for the present study with information on localities, voucher specimens, and GenBank accession numbers for nrDNA ITS.

Samples	Taxon	Locality	Coordinates	Collectors	Voucher specimens	GenBank acc.no.
A1252	*A. fucensis*	I, Abruzzo, M. Annamunna, Collelongo, 1038 m.	41°55′21″ N – 13°38′8″ E	*Bartolucci and Conti s.n.*	APP 66208	OP537844
A1250	*A. volgensis*	RUS, Tambow, Mordowo, Streletzkaja.	-	*Sukhorukov s.n.*	B 10 0210009	OP537841
A1251	*A. volgensis*	ROM, Constanta, Cotu Văii, Valea Mare, 85 m.	43°48′29″ N – 28°20′08″ E	*Negrean s.n.*	B 10 0210277	OP537842 (a) OP537843 (b)
A1267	*A. volgensis*	RUS, Bashkortostan, Baimak, Semenovskoe, 618 m.	52°31′00″ N – 58°17′11″ E	*Kočí, Tichý and Horsák 2007/021*	BRNU 591605	
A1268	*A. volgensis*	RUS, Bashkortostan, Abzanovo, Verkhnii Muinak, 327 m.	52°04′11″ N – 56°44′16″ E	*Chytrý, Otýpková and Lososová 2007/022*	BRNU 591606	
A1269	*A. volgensis*	KAZ, Karaganda, Abaisk Topar, 624 m.	49°30′25″ N – 72°54′18″ E	*Danihelka, Dřevojan and Kubesová 2014/786*	BRNU 653944	
A1270	*A. volgensis*	KAZ, Akmola, Ereymentau, 454 m.	51°34′30″ N – 73°08′18″ E	*Danihelka, Dřevojan and Kubesová 2014/392*	BRNU 653564	

A1271	*A. volgensis*	KAZ, Akmola, Ereymentau, 446 m.	51°34′17″ N – 73°08′11″ E	*Chytrý & Hájek 2014/393*	BRNO 653565	
A1272	*A. volgensis*	ROM, Constanta, Murfatlar, Fantanita.	44°09′34″ N – 28°23′28″ E	*Nicoară s.n.*	APP 66217	
A1273	*A. volgensis*	ROM, Constanta, Cotu Văii.	43°49′45″ N – 28°21′14″ E	*Nicoară s.n.*	APP 66214	
A1274	*A. volgensis*	ROM, Constanta, Cotu Văii.	43°48′30″ N – 28°20′07″ E	*Nicoară s.n.*	APP 66216	
A1275	*A. volgensis*	ROM, Cluj, Fanatele Clujului.	46°48′55″ N – 23°39′35″ E	*Nicoară s.n.*	APP 66215	
